# Subaortic Membrane Mimicking Hypertrophic Cardiomyopathy in the Setting of Mitral Valve Infective Endocarditis

**DOI:** 10.7759/cureus.41129

**Published:** 2023-06-29

**Authors:** Aws Polina, Woosun Kang, Rajendra P Shah, M. Chadi Alraies

**Affiliations:** 1 Internal Medicine, Wayne State University Detroit Medical Center, Detroit, USA; 2 Cardiology, Wayne State University Detroit Medical Center, Detroit, USA; 3 Internal Medicine, Vassar Brothers Medical Center, Poughkeepsie, USA

**Keywords:** cerebral mycotic aneurysm, acute subarachnoid hemorrhage, subaortic valvular membrane, non obstructive hypertrophic cardiomyopathy, infective endocarditis

## Abstract

Infective endocarditis (IE) can present in a wide spectrum of systemic signs and symptoms. Here we report a case of a patient who presented with a headache. Upon further investigation, the patient was found to have mitral valve IE. This likely led to a subarachnoid hemorrhage secondary to a ruptured mycotic aneurysm. In this case report, we emphasize the importance of noticing early neurological signs of IE even when initial imaging is negative for aneurysmal formation. Further, this patient had a subaortic membrane (SAoM) mimicking the sonographic appearance of hypertrophic obstructive cardiomyopathy. Remarkably, SAoM is usually associated with aortic valvular pathology; however, this patient unusually presented with mitral valve involvement.

## Introduction

The clinical features of infective endocarditis (IE) were identified as early as the 16th century by the French physician Jean François Fernel. A few centuries later, Sir William Osler further described the physical exam findings associated with IE and shone a light on the disease through a series of lectures [1]. Now we identify IE as a sequela of symptoms that begins with the infection of previously damaged cardiac endocardium surface and valves [2]. Injury to the endocardium endothelium occurs via shearing forces created by turbulent or high-pressure jet flow. When the endocardium is damaged, collagen and tissue factors are exposed, which consequently results in fibrin and platelet deposition leading to thrombus formation. Microbial pathogens can then adhere to this thrombus, resulting in the formation of septic emboli. 

Similar to the mechanism of endocardium injury in IE, the formation of a subaortic membrane (SAoM) is associated with high-velocity turbulent blood flow that occurs due to variation in the left ventricular outflow tract (LVOT) anatomy [3]. SAoM is usually composed of either a thin, discrete fibrous membrane or a circular fibromuscular membrane located in the LVOT. As a result, this variation in anatomy can increase the risk of IE. Both clinically and sonographically, SAoM can mimic hypertrophic obstructive cardiomyopathy (HOCM) as both can cause LVOT obstruction. Systolic anterior motion (SAM) of the mitral valve used to be considered a highly specific feature of HOCM. However, throughout recent years, SAM of the mitral valve has been associated with other structural cardiac abnormalities including SAoM [4-6]. 

The clinical presentation of IE varies and encompasses a wide spectrum of systemic signs and symptoms. The most common presenting symptoms of IE are fever and new cardiac murmur. In contrast, splinter hemorrhages, Janeway lesions, Osler nodes, and Roth spots are highly specific but infrequent clinical presentations of IE [7]. Neurological complications occur in 15 to 30% of IE cases [8]. The ruptured mycotic aneurysm is a subset of hemorrhagic stroke that can present as a rare complication of IE. A mycotic aneurysm occurs when septic emboli dislodge to a distal artery bifurcation leading to infection and necrosis of the vessel wall layers. As a result, the destruction of the muscularis and adventitial layers causes dilatation of the vessel wall and aneurysmal formation. Mycotic aneurysms can involve any vessel including intracranial vessels and when ruptured, they are associated with high morbidity and mortality rates. Ruptured intracranial aneurysms can present as either intracerebral hemorrhage or subarachnoid hemorrhage (SAH). However, about 15% of patients with nontraumatic SAH do not have any vascular lesions and are called non-aneurysmal SAH [9]. 

## Case presentation

**Figure 1:**
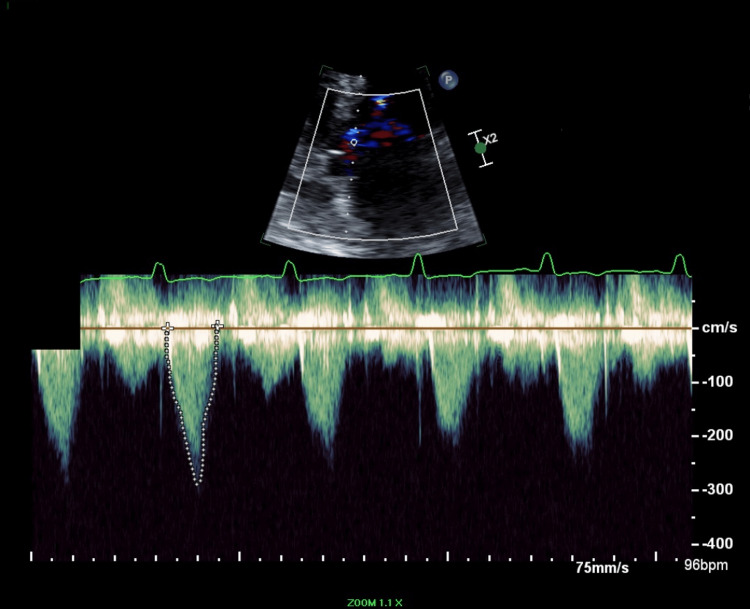
Transthoracic echocardiography image Late peaking continuous wave Doppler for left ventricular outflow tract, which suggests dynamic obstruction with evidence of chordal systolic anterior motion of the mitral valve.

**Figure 2:**
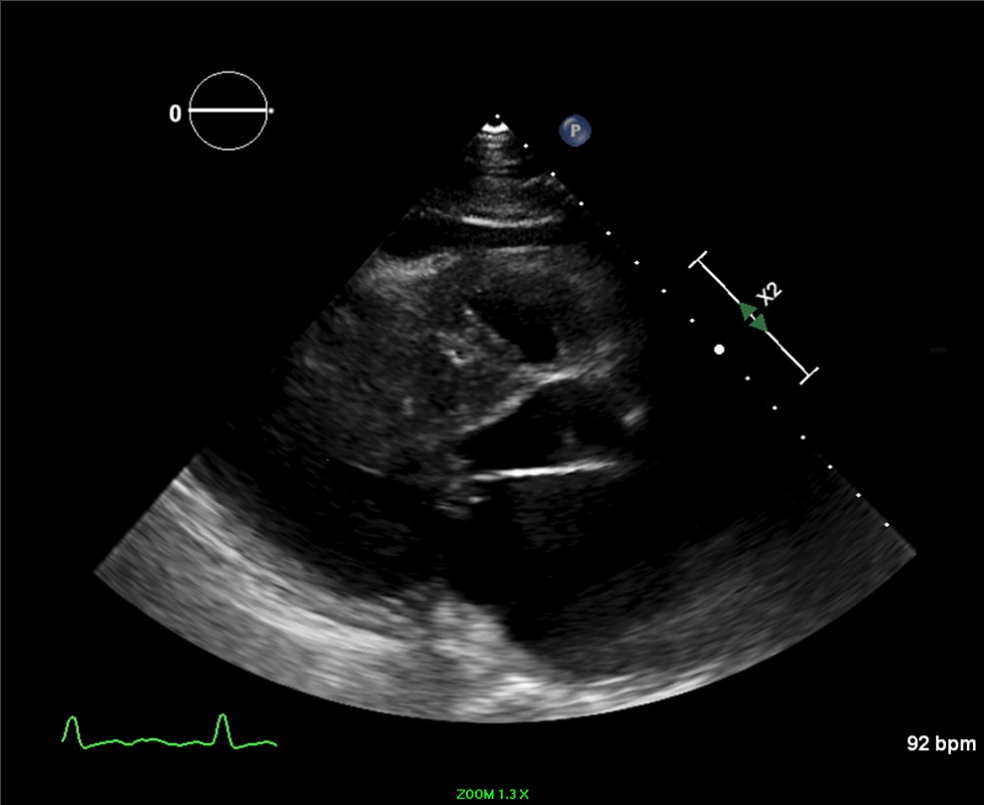
TTE parasternal long axis view TTE: Transthoracic echocardiogram Parasternal long axis view shows chordal systolic anterior motion and thickened anterior mitral valve leaflet.

A 32-year-old woman with a past medical history of recurrent sinusitis and right knee pain presented to the hospital with a subacute headache. Approximately a month before the presentation, the patient was treated with a five-day course of amoxicillin for a presumed bacterial sinus infection. She showed temporary improvement in her symptoms but then started having constant, generalized headaches. The headaches progressively worsened in frequency and severity and were associated with chills, night sweats, weight loss, dizziness, and photophobia. On presentation to the hospital, she was febrile with a temperature of 38.6°C and tachycardic but otherwise hemodynamically stable. The physical examination was remarkable for a high-pitched, systolic murmur auscultated in the left sternal border and cardiac apex. The patient had no focal neurological deficits and the cranial nerves exam was grossly intact. The extremities were examined and showed no cutaneous lesions, tenderness to palpation, or erythema. Further, there were no splinter hemorrhages noted on the nail beds and oral examination was unremarkable. Laboratory studies were significant for leukocytosis and elevated inflammatory markers. Blood cultures were positive for *Streptococcus mitis oralis*, which was amoxicillin-resistant. Given the bacteremia and the new cardiac murmur, a transthoracic echocardiogram (TTE) was obtained. TTE noted increased left ventricular (LV) wall thickness with mild dynamic obstruction and severe hypertrophy in the LVOT. Additionally, the mitral valve showed moderate to severe regurgitation, SAM (Figure 1), and a 10x10 mm vegetation on the anterior leaflet (Figure 2). Therefore, a transesophageal echocardiogram (TEE) was obtained to further characterize the valvular vegetation. TEE revealed LVOT obstruction as well as two large vegetations located in the posterior and anterior mitral valve leaflets measuring 18x10 mm and 16x10 mm, respectively. Given these findings, the patient was diagnosed with IE and treated with ceftriaxone. 

To evaluate the patient’s headache, computed tomography (CT) and magnetic resonance imaging (MRI) of the head were obtained and showed a subarachnoid hemorrhage (SAH) along the right Sylvian fissure and precentral sulcus. A head and neck CT angiogram (CTA) was also performed and noted no evidence of aneurysm, arteriovenous (AV) malformation, or AV fistula. Later during the hospitalization, she developed transient bilateral vision loss and was found to have bilateral Roth spots on examination. Repeat head imaging showed sulcal hemosiderosis reflecting chronic sequela of SAH as well as three chronic microhemorrhages in the lobar distribution. Given the history of IE and the distribution of microhemorrhages, a ruptured distal mycotic aneurysm was highly suspected. The SAH size progressively decreased and the patient underwent bioprosthetic mitral valve placement. During cardiac surgery, a discrete SAoM was also identified. Initial echocardiography findings were suggestive of LVOT obstruction, which were likely secondary to SAoM. The SAoM was sampled and the pathology was consistent with a fibrous membrane. The post-surgical course was uncomplicated and the patient was discharged to a rehab facility.

## Discussion

The initial sonographic findings for this patient were suggestive of HOCM given the dynamic LVOT obstruction and the SAM of the mitral valve. Nonetheless, surgically she was found to have a discrete fibrous membrane in the LVOT. SAoM can cause LVOT narrowing and may imitate HOCM echocardiographic findings. However, in SAoM, the LVOT obstruction is usually fixed compared to a variable and dynamic obstruction seen in HOCM. Additionally, changes in hemodynamics lead to variation in LVOT obstruction in HOCM but have no significant impact on SAoM. HOCM and SAoM can be differentiated via TTE or TEE with Doppler and pressure gradients. This distinction is important as their pathophysiology and management differ. Furthermore, SAoM has been linked with IE and the expected location of the vegetation is usually the aortic valve and/or SAoM itself [10]. However, this patient unusually presented with mitral valve IE. The pathophysiology of how this phenomenon occurs is unclear, but it is likely related to the turbulent flow created by SAoM leading to abnormal valvular motion and shearing forces of the mitral valve leaflets.

This patient’s presentation of IE was associated with a unique clinical neurological manifestation. Further, the etiology of the SAH was thought to be secondary to a ruptured mycotic aneurysm given its distribution on imaging. However, the initial CTA did not show any aneurysmal formation. Some studies have noted that up to 24% of patients with SAH have initial negative angiography, but later were found to have an aneurysm on repeat imaging [11]. There is no consensus on the need and timing for repeat brain imaging and the decision should be based on the patient’s clinical presentation and the presence of complications. Therefore, it is essential to notice early signs of IE and its neurological complications even when initial studies are negative for mycotic aneurysmal formation. Furthermore, the management approach for these patients should be individualized as the risks and benefits of valvular replacement should be meticulously considered in the setting of intracranial bleeding. 

## Conclusions

In conclusion, this case highlights how SAoM may cause LVOT narrowing and SAM, which can mimic HOCM imaging findings. Therefore, attention to detail such as the characteristic of the LVOT obstruction can help distinguish these two entities. Furthermore, the turbulent flow created by the LVOT narrowing and SAM may predispose individuals to IE as exemplified by our patient’s presentation. Lastly, the clinical presentation of IE can vary greatly, and attention to neurological manifestations of IE, although an uncommon initial presentation, can lead to early diagnosis and management of IE.
